# Two-Component Signal Transduction System CBO0787/CBO0786 Represses Transcription from Botulinum Neurotoxin Promoters in *Clostridium botulinum* ATCC 3502

**DOI:** 10.1371/journal.ppat.1003252

**Published:** 2013-03-28

**Authors:** Zhen Zhang, Hannu Korkeala, Elias Dahlsten, Elina Sahala, John T. Heap, Nigel P. Minton, Miia Lindström

**Affiliations:** 1 Department of Food Hygiene and Environmental Health, Faculty of Veterinary Medicine, University of Helsinki, Helsinki, Finland; 2 Clostridia Research Group, Centre for Biomolecular Sciences, University of Nottingham, Nottingham, United Kingdom; University of Illinois, United States of America

## Abstract

Blocking neurotransmission, botulinum neurotoxin is the most poisonous biological substance known to mankind. Despite its infamy as the scourge of the food industry, the neurotoxin is increasingly used as a pharmaceutical to treat an expanding range of muscle disorders. Whilst neurotoxin expression by the spore-forming bacterium *Clostridium botulinum* appears tightly regulated, to date only positive regulatory elements, such as the alternative sigma factor BotR, have been implicated in this control. The identification of negative regulators has proven to be elusive. Here, we show that the two-component signal transduction system CBO0787/CBO0786 negatively regulates botulinum neurotoxin expression. Single insertional inactivation of *cbo0787* encoding a sensor histidine kinase, or of *cbo0786* encoding a response regulator, resulted in significantly elevated neurotoxin gene expression levels and increased neurotoxin production. Recombinant CBO0786 regulator was shown to bind to the conserved −10 site of the core promoters of the *ha* and *ntnh*-*botA* operons, which encode the toxin structural and accessory proteins. Increasing concentration of CBO0786 inhibited BotR-directed transcription from the *ha* and *ntnh*-*botA* promoters, demonstrating direct transcriptional repression of the *ha* and *ntnh*-*botA* operons by CBO0786. Thus, we propose that CBO0786 represses neurotoxin gene expression by blocking BotR-directed transcription from the neurotoxin promoters. This is the first evidence of a negative regulator controlling botulinum neurotoxin production. Understanding the neurotoxin regulatory mechanisms is a major target of the food and pharmaceutical industries alike.

## Author Summary

Botulinum neurotoxin produced by the spore-forming bacterium *Clostridium botulinum* is the most poisonous biological substance known to mankind. By blocking neurotransmission, the neurotoxin causes a flaccid paralysis called botulism which may to lead to death upon respiratory muscle collapse. Despite its infamy as the scourge of the food industry, the neurotoxin is attracting increasing interest as a pharmaceutical to treat an expanding range of muscle disorders. Whilst neurotoxin production by *C. botulinum* appears tightly regulated, to date only positive regulatory elements, thus enhancing the neurotoxin production, have been implicated in this control. The identification of negative regulators, responsible for down-tuning the neurotoxin synthesis, has proven to be elusive, but would offer novel approaches both for the production of safe foods and for the development of therapeutic neurotoxins. Here, we report a two-component signal transduction system that negatively regulates botulinum neurotoxin production. Understanding the neurotoxin regulatory mechanisms is a major target of the food and pharmaceutical industries alike.

## Introduction

Botulinum neurotoxins are the most poisonous biological substances known to mankind. The neurotoxins are metalloproteases which block neurotransmission in cholinergic nerves [1], [2] in humans and animals to cause botulism, a potentially lethal flaccid paralysis. Botulinum neurotoxins are produced by vegetative cultures of the anaerobic spore-forming bacterium *Clostridium botulinum* which is widespread in the environment. The neurotoxins can enter the victim's body through intoxication with food or drink, or they can be produced from spores germinating and growing into active cultures *in vivo*, most likely in the gut of small babies with poorly developed gut microflora or in deep wounds. Despite their infamy, botulinum neurotoxins attract increasing interest as a pharmaceutical to treat an expanding range of muscular and other disorders [3], [4], such as torticollis, focal dystonia, inappropriate contraction of gastrointestinal sphincters, eye movement disorders, hyperhidrosis, migraine [5], genitourinary disorders [6], and even cancer [7]. Indications in cosmetic surgery are well known.

**Figure 1:**
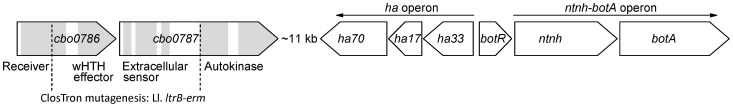
Schematic representation of the TCS CBO0787/CBO0786 and neurotoxin loci in *C. botulinum* ATCC 3502. The neurotoxin operons are indicated with arrows. Predicted TCS domains are marked with gray color and the corresponding functions are listed under each gene. Insertional sites of ClosTron mutagenesis in *cbo0786* encoding a response regulator and *cbo0787* encoding a sensor histidine kinase are indicated with dashed lines.

Seven antigenically distinct toxin types (A to G), and several subtypes therein, have been described [8]–[12]. Type A1 neurotoxins are the best characterized, a consequence both of their frequent involvement in human botulism worldwide and of their greater potency and, therefore, suitability for therapeutics [13]. Botulinum toxins are produced as a complex containing the neurotoxin itself and one or more non-toxic auxiliary proteins that protect the neurotoxin from environmental stress and assist in absorption [14]. Type A1 toxins are complexed with the non-toxic non-hemagglutinating (NTNH) protein and three hemagglutinins (HA17, HA33 and HA70) [15]–[18]. A typical A1-type gene cluster is transcribed in two operons, namely the *ntnh*-*botA* and *ha* operons [19] (Figure 1). Both operons have consensus −10 and −35 core promoter sequences, which are recognized by the alternative sigma factor BotR, directing RNA polymerase (RNAP) to transcribe the two operons [20]. The gene encoding BotR is located between the two operons within the neurotoxin gene cluster.

Botulinum neurotoxin production is affected by the availability of certain nutrients [21]–[23] and is associated with transition from late-exponential to early-stationary phase cultures. A peak in the level of neurotoxin gene cluster expression in late-exponential to early-stationary phase cultures [19], [24] suggests that neurotoxin production is tightly regulated. To date only positive regulatory elements have been implicated in this control. These include the participation of BotR [25] and an Agr quorum sensing system [26]. The identification of negative regulators of botulinum neurotoxin production has until now proved to be elusive.

Two-component signal transduction systems (TCS) are conserved in bacteria and differentially specialized to control a range of cellular events in response to environmental stimuli. The histidine kinases sense cellular or environmental signals through the N-terminus of their sensor domains. This interaction leads to autophosphorylation at a histidine residue in their C-terminus and the subsequent activation of their cognate response regulator present in the cytosol by transmission of the phosphoryl group to an N-terminal aspartate residue of the response regulator and further to the C-terminal output domain. Response regulators possess DNA-binding activity, ultimately resulting in a specific response in the expression of their target genes. The individual roles of most TCSs in *C. botulinum* are not known, but their involvement in control of virulence in other pathogenic bacteria has been demonstrated [27]. Antisense mRNA inhibition of genes encoding three TCSs caused decreased neurotoxin production in *C. botulinum* type A strain Hall [28], suggesting these TCSs may play a role in positive control of neurotoxin synthesis. The model strain *C. botulinum* ATCC 3502 (Group I, type A) [29] encodes 29 putative TCSs and a set of orphan histidine kinases and response regulators [30]. One of the intact TCSs, CBO0787/CBO0786 (Figure 1), shares over 90% amino acid identity with other *C. botulinum* Group I strains and in many strains is located in the vicinity of the neurotoxin genes (3.6 to 24 kilobases [kb] up- or downstream of the toxin genes). Here we show that the TCS CBO0787/CBO0786 negatively regulates botulinum neurotoxin gene expression. Understanding the regulatory mechanisms that control the production of botulinum neurotoxin is a major target of the food and pharmaceutical industries.

## Results

### The *cbo0787* and *cbo0786* genes are down-regulated at transition into stationary growth phase

**Figure 2:**
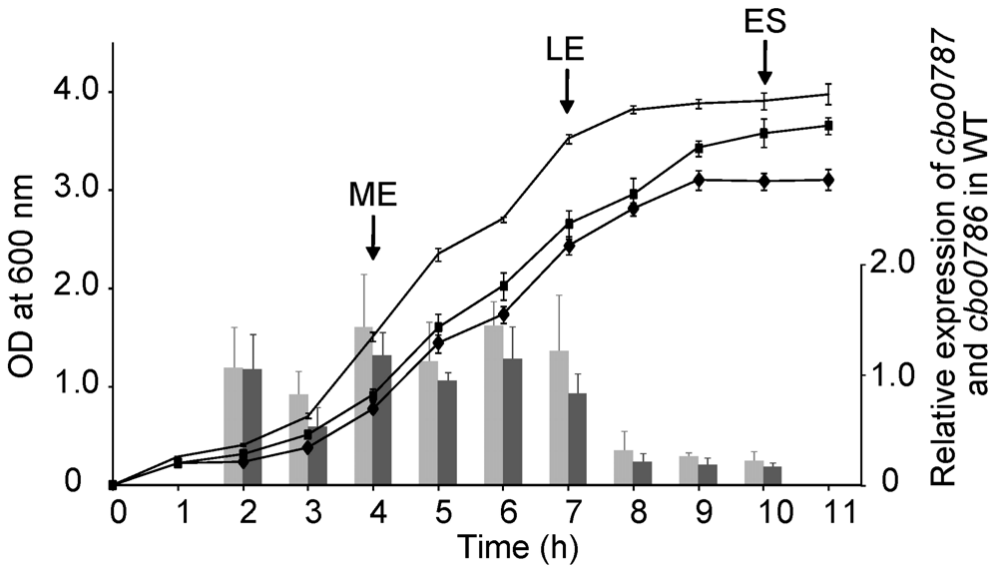
Expression of *cbo0787* and *cbo0786* in *C. botulinum* ATCC 3502 wild type (WT) and the growth curves of WT and the *cbo0787* and *cbo0786* mutants. Quantitative RT-PCR analysis of relative *cbo0787* (light grey bars) and *cbo0786* (dark grey bars) transcript levels in WT. Target gene expression was normalized to 16S *rrn* and calibrated to 2-hour time point. Growth of WT (line without symbols), *cbo0787* mutant (square) and *cbo0786* mutant (diamond) in tryptose-peptone-glucose-yeast extract medium. Arrows indicate sampling at mid-exponential (ME), late exponential (LE) and early stationary (ES) growth phases. Error bars represent standard deviations of three replicate measurements.

We used quantitative reverse transcription PCR (qRT-PCR) to measure the relative expression of *cbo0787* and *cbo0786* during the growth of *C. botulinum* Group I type A strain ATCC 3502, which is the most widely used model strain for genetic studies in *C. botulinum* [26], [31], [32]. The relative transcription levels of *cbo0787* and *cbo0786* followed an identical pattern, suggesting that the two genes are co-transcribed (Figure 2). In relation to growth, *cbo0787* and *cbo0786* were expressed at a relatively constant level throughout the logarithmic growth phase, and were down-regulated at the transition into stationary phase (Figure 2).

### Mutation of *cbo0787* and *cbo0786*

**Figure 3:**
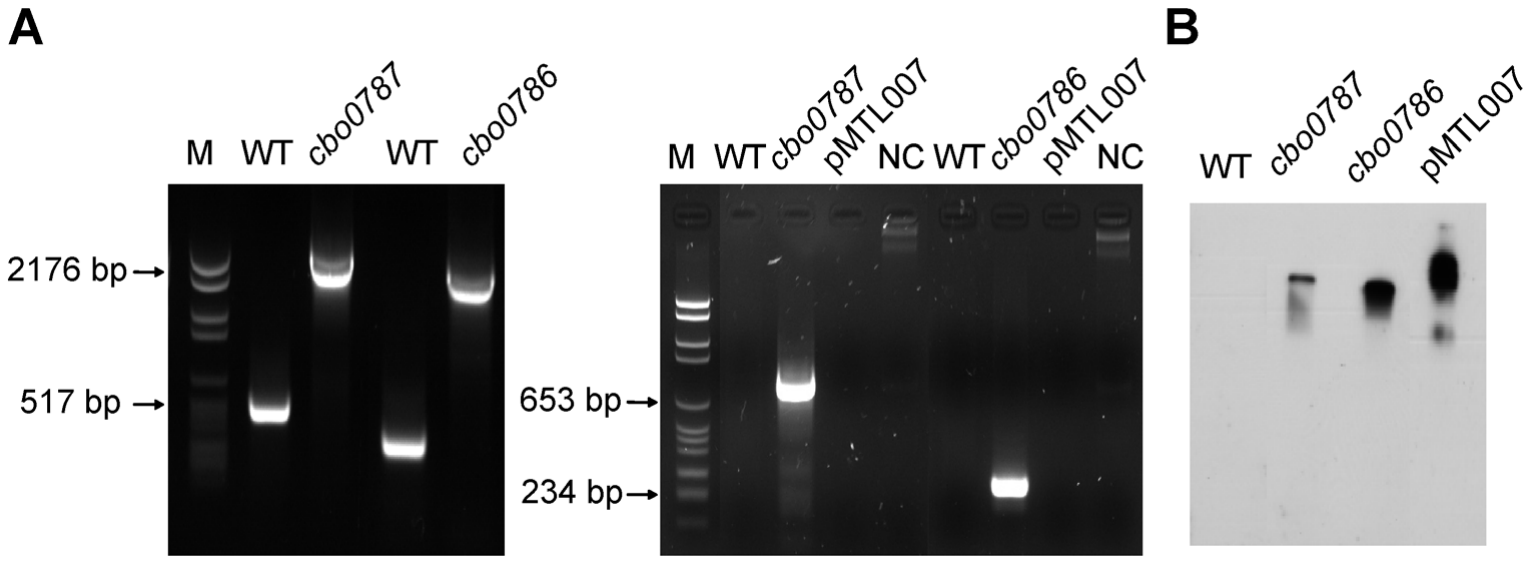
Insertional inactivation of *cbo0787* and *cbo0786*. (A) PCR analysis of mutations. Ll.LtrB intron insertion was detected with primers flanking the insertional sites yielding a 2.3-kb PCR product in *cbo0787* mutant and a 2.2-kb product in *cbo0786* mutant (left), and with a gene-specific primer and the intron-binding primer EBS universal yielding a 785-bp product in the *cbo0787* mutant and a 260-bp product in the *cbo0786* mutant (right). (B) Southern blot analysis of HindIII digested genomic DNA from WT and *cbo0787* and *cbo0786* mutants with intron-specific probe.

To address the role of CBO0787/CBO0786, we constructed single, insertional inactivation mutations in *cbo0787* or *cbo0786* using the ClosTron tool [33]. Single insertion of the group II intron from pMTL007 into the desired sites in *cbo0787* or *cbo0786* (Figure 1) was confirmed by PCR (Figure 3A) and Southern blotting (Figure 3B). Consecutive cultures showed the mutants to be erythromycin resistant and stable. No significant difference between the growth of the TCS mutants and the ATCC 3502 wild-type strain (WT) were observed (Figure 2), and the log cell counts per ml of WT and the *cbo0787* and *cbo0786* mutant cultures at early stationary phase (10 hours) were 8.9, 9.0 and 8.8, respectively.

### The *cbo0787* or *cbo0786* mutants show induced expression of the neurotoxin cluster genes

**Figure 4:**
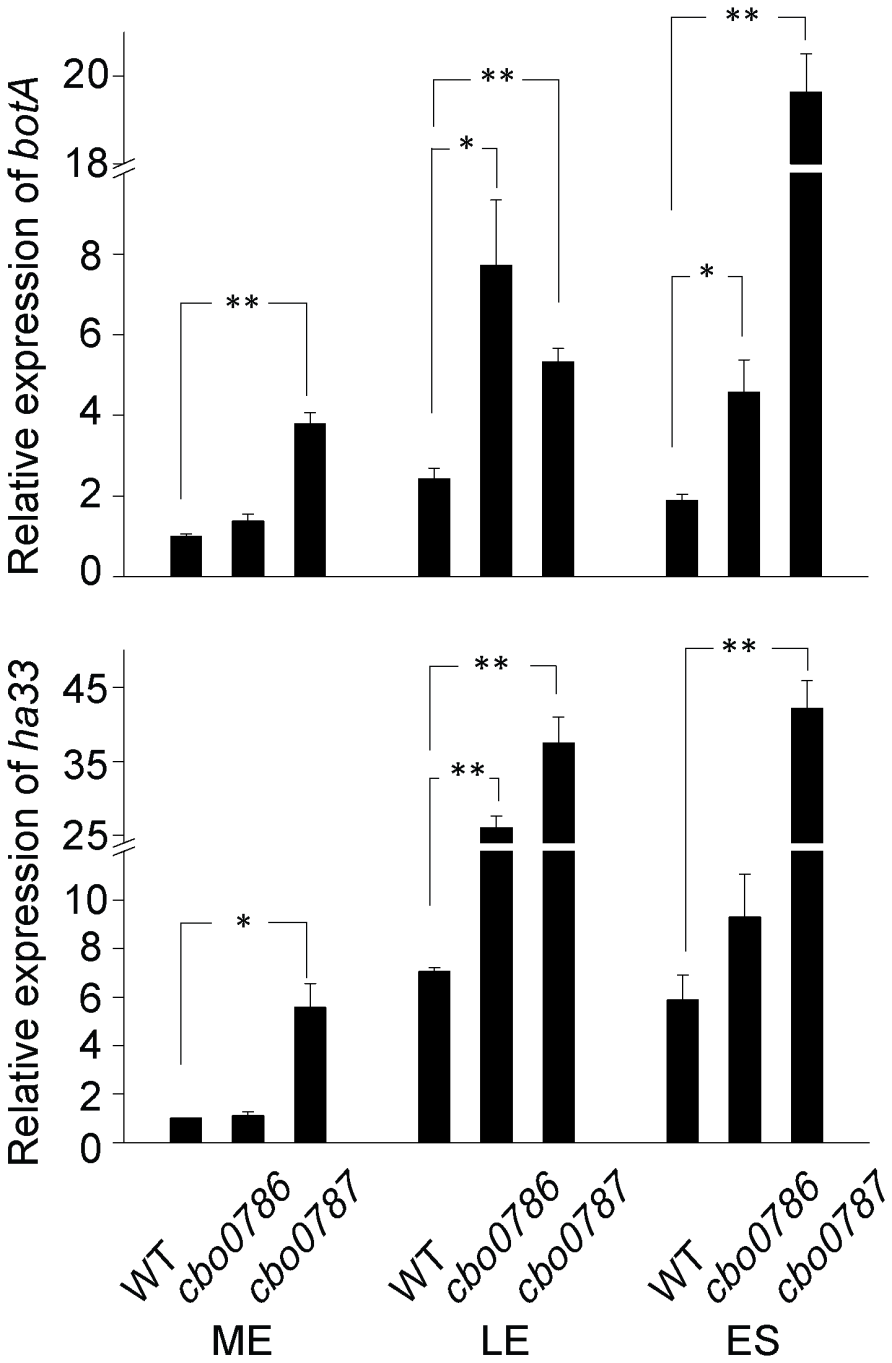
Disruption of *cbo0787* or *cbo0786* increased the expression of neurotoxin genes. Quantitative RT-PCR analysis of relative *botA* and *ha33* transcript levels in WT and the *cbo0786* and *cbo0787* mutants. RNA was isolated from cells at mid-exponential (ME), late exponential (LE) and early stationary (ES) growth phases. Target gene expression was normalized to 16S *rrn* and calibrated to WT at ME. Error bars represent standard deviations of three replicate measurements. Statistical significance of differences between WT and each mutant is indicated with p-values (*, *P*<0.05; **, *P*<0.01).

We used qRT-PCR to measure the relative expression of *botA* encoding botulinum neurotoxin type A and *ha33* encoding one of the three haemagglutinins in WT and the two TCS mutants at mid-exponential, late exponential and early stationary growth phases. The two genes were selected to represent the two operons present in the neurotoxin gene cluster, and the three time points used have been shown to associate with induction and repression of the neurotoxin gene expression [19], [34], while later time points typically involve other cellular events, such as sporulation or lysis, and were thus not considered relevant. As expected, the WT levels of *botA* and *ha33* expression peaked at late exponential or early stationary growth phases (Figure 4). Strikingly, the *cbo0787* mutant had a maximum of 10.4-fold (*P*<0.01) and 8.0-fold (*P*<0.01) higher relative *botA* and *ha33* expression levels, respectively, than the WT (Figure 4), the most prominent differences being observed at early stationary growth phase. This suggests that the histidine kinase CBO0787 is required to sense an as yet unidentified signal for the efficient ‘switch-off’ of the neurotoxin gene expression at transition into stationary growth phase.

The *cbo0786* mutant also showed significantly increased *botA* and *ha33* expression levels in relation to WT (Figure 4). A maximum of 2.4-fold (*P*<0.05) induction for *botA* at early stationary phase and 3.8-fold (*P*<0.01) induction for *ha33* at late exponential growth phase were measured. These data suggest that CBO0786 negatively regulates neurotoxin gene expression, either directly or indirectly.

### The *cbo0787* or *cbo0786* mutants show induced neurotoxin production

**Figure 5:**
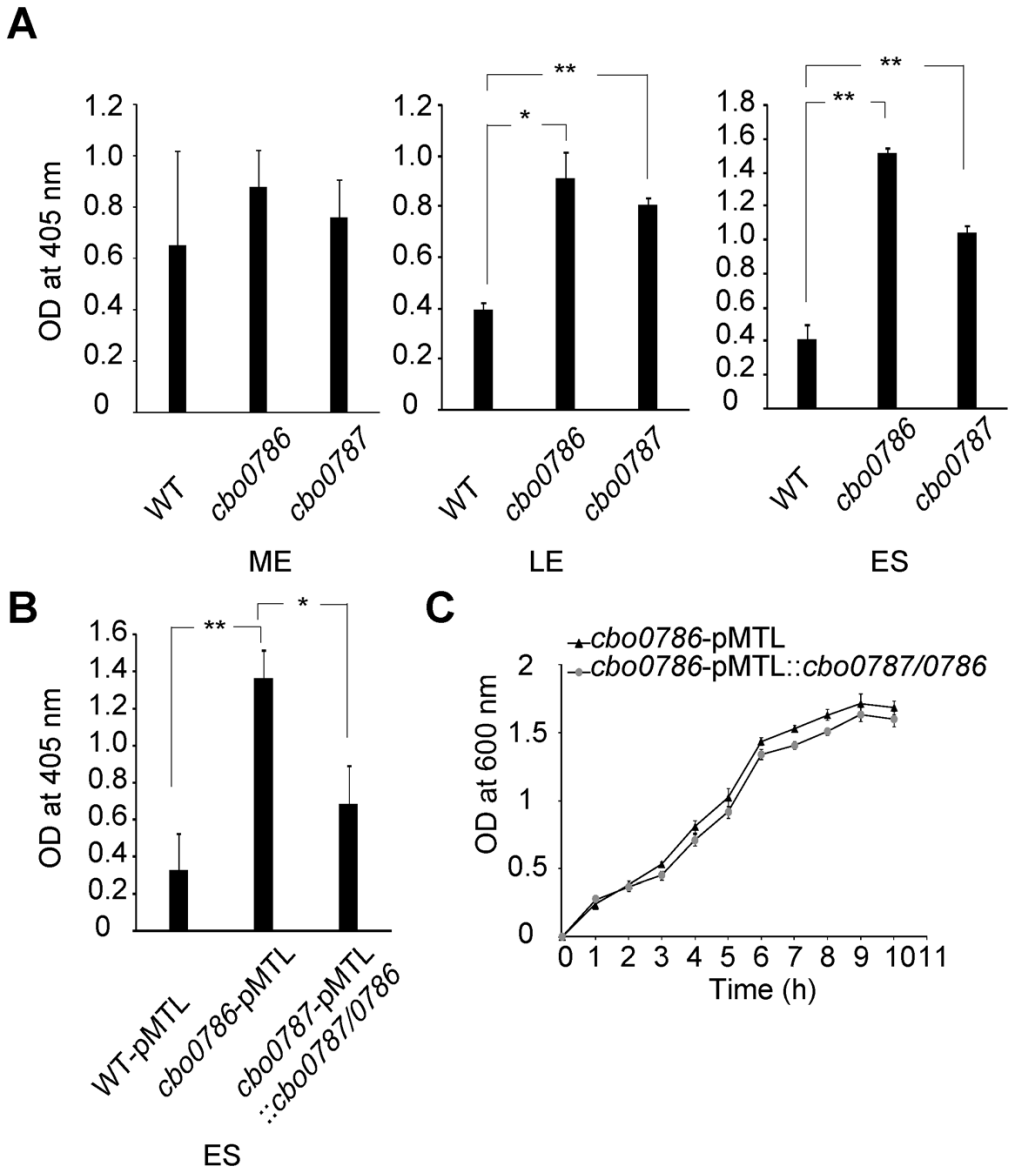
Neurotoxin ELISA to demonstrate elevated neurotoxin production by *cbo0787* and *cbo0787* mutants. (A) ELISA analysis of botulinum neurotoxin in culture supernatants of WT and the *cbo0786* and *cbo0787* mutants at mid-exponential (ME), late exponential (LE) and early stationary (ES) growth phases. The samples were diluted 1∶10 at ME, 1∶20 at LE, and 1∶30 at ES. (B) ELISA analysis of botulinum neurotoxin in culture supernatants of the vector-only control strains WT-pMTL and *cbo0786*-pMTL, and the complemented strain *cbo0786*-pMTL::*cbo0787*/*0786* at ES. All samples were diluted 1∶30. (C) Growth curve of *cbo0786*-pMTL and *cbo0786*-pMTL::*cbo0787*/*cbo0786*. Error bars represent standard deviations of three replicate measurements. Statistical significance of differences between WT and the two mutants is indicated with p-values (*, *P*<0.05; **, *P*<0.01).

To confirm that our findings at the transcription level apply to protein level, we analyzed the relative amounts of botulinum neurotoxin in the WT and mutant culture supernatants collected at mid-exponential, late exponential and early stationary growth phases. Measurements were made using an enzyme-linked immunosorbent assay (ELISA). At mid-exponential growth phase, neurotoxin production was slightly but not significantly increased in the *cbo0787* and *cbo0786* mutant cultures compared to WT culture (Figure 5A). At late exponential and early stationary growth phases, neurotoxin production was significantly increased (2.1 to 3.7-fold higher OD_405_ readings, *P*<0.05) in both the *cbo0787* and *cbo0786* mutant cultures relative to WT culture (Figure 5A). To validate this result, we complemented the *cbo0786* mutation by introducing pMTL::*cbo0787*/*0786*, containing the TCS genes and their putative native promoter, into the *cbo0786* mutant and showed that neurotoxin levels in early-stationary phase cultures were restored to the vector-only control (WT-pMTL) level (*P*<0.05, Figure 5B). No difference was observed in growth between the *cbo0786*-pMTL control and the complemented strain (Figure 5C).

### The CBO0786 response regulator binds to neurotoxin promoters

**Figure 6:**
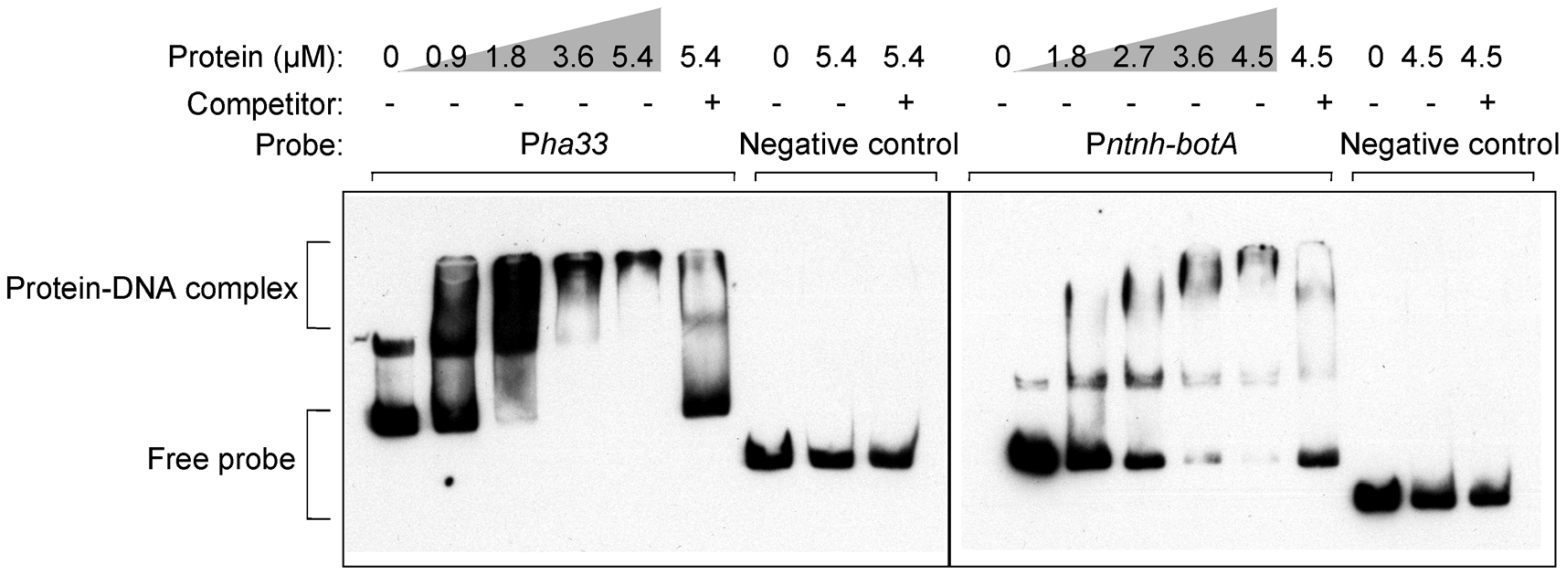
CBO0786 binds to botulinum neurotoxin promoters *in vitro*. EMSA showing CBO0786 binding to P*ha33* probe (left panel) and P*ntnh-botA* probe (right panel). Specificity was confirmed using 200-fold molar excess of non-labeled competitor DNA and a random gene fragment as negative control.

**Figure 7:**
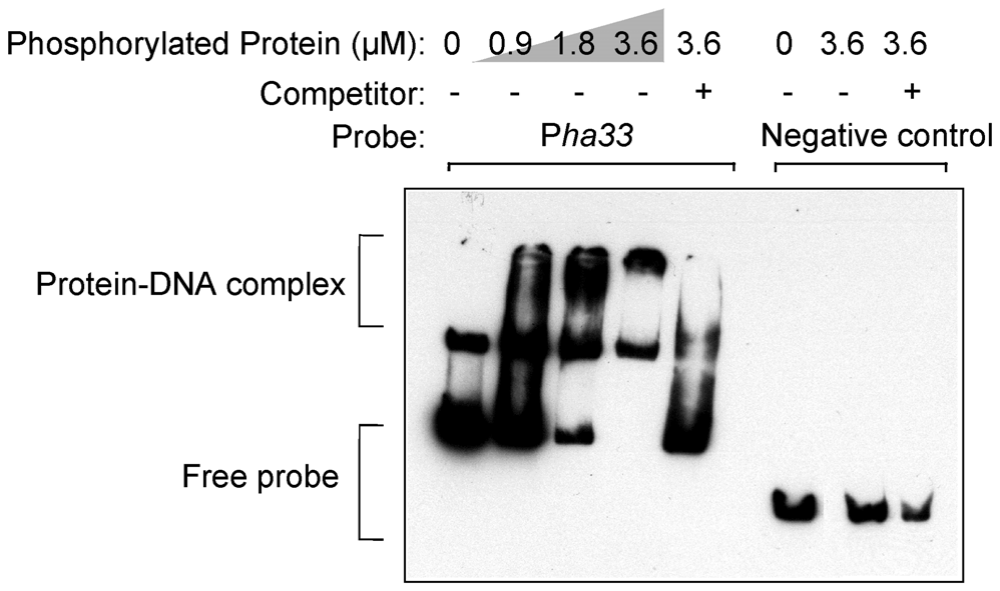
*In vitro* phosphorylated CBO0786 binds to P*ha33* probe *in vitro*. EMSA showing phosphorylated CBO0786 binding to P*ha33* probe. Specificity was confirmed using 200-fold molar excess of non-labeled competitor DNA and a random gene fragment as negative control.

To test the hypothesis that the TCS response regulator CBO0786 is a transcriptional repressor of the neurotoxin gene cluster in ATCC 3502, we examined the binding of the recombinant CBO0786 protein to probes that encompassed the intergenic region between *ha33* and *botR* containing the promoter of the *ha* operon [35] (P*ha33* probe), or the intergenic region between *botR* and *ntnh* containing the promoter of the *ntnh*-*botA* operon [35] (P*ntnh-botA* probe), by electrophoretic mobility shift assay (EMSA). CBO0786 caused a shift in the mobility of both probes, although its binding affinity to the P*ntnh-botA* probe appeared somewhat lower than to the P*ha33* probe (Figure 6). The specific nature of binding was further confirmed by disappearance of both protein-DNA complexes using competition with a 200-fold excess of unlabeled probe. Moreover, no electrophoretic shift was observed with the negative control probe (Figure 6). EMSA with recombinant CBO0786 phosphorylated by acetyl phosphate yielded a similar shift (Figure 7), indicating that phosphorylation is not essential for CBO0786 binding to DNA *in vitro*. These results suggest that CBO0786 recognizes and binds to the promoter regions of the *ha* and the *ntnh*-*botA* operons.

**Figure 8:**
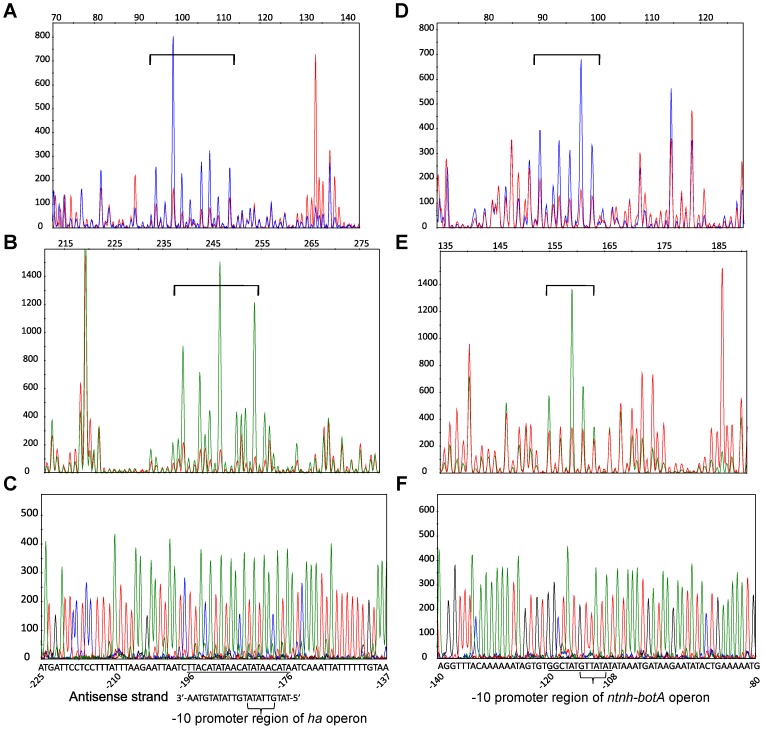
CBO0786 binds to the core promoter −10 region of the *ha* and *ntnh*-*botA* operons. DNase I footprinting analysis of 5′-6-FAM labeled sense strand (A, D) and 5′-HEX labeled antisense strand (B, E) of P*ha33* probe (A, B) and P*ntnh-botA* probe (D, E). Comparison of DNase I digestion in the absence (blue peaks in A and D, green peaks in B and E) or with 10 µM of CBO0786 (red peaks). Protection regions are indicated by square brackets. Protection regions are underlined in sequencing electropherograms of P*ha33* probe (C) and P*ntnh-botA* probe (F) in the sense strand. The consensus −10 regions of the *ha* and *ntnh-botA* promoters are indicated.

To understand the DNA-binding specificity of CBO0786, we identified its binding sites using DNase I footprinting and fluorescently end-labeled P*ha33* and P*ntnh-botA* probes (Figure 8). With the P*ha33* probe, the region protected by CBO0786 (−51 bp to −31 bp upstream of *ha33* and −196 bp to −176 bp upstream of *botR*) was present in both strands (Figures 8A and 8B). Greater protection by CBO0786 was observed in the antisense strand containing the promoter of the *ha* operon [35], suggesting that the antisense sequence (TATGTTATATGTTATATGTAA, Figure 8C) is the major CBO0786 binding site. Interestingly, the core promoter −10 region (GTTATA) of the *ha* operon, recognized by the alternative sigma factor BotR [20], appeared as a direct repeat in the CBO0786 binding site, suggesting that CBO0786 represses transcription of the *ha* operon by binding to its core promoter.

In the P*ntnh-botA* probe, a site of CBO0786 protection similar to that observed with the P*ha33* probe was evident (Figures 8D and 8E). Both strands contained the same protection site (GGCTATGTTATAT) (−120 bp to −108 bp upstream of *ntnh*, Figure 8F). Accordingly, the −10 region (GTTATA) of the *ntnh-botA* operon core promoter was presented in the binding region, but in one copy only. In accordance with the EMSA analysis, the binding affinity of CBO0786 to the *ntnh-botA* promoter appeared lower than to the promoter of the *ha* operon.

### The CBO0786 response regulator represses *in vitro* transcription from neurotoxin promoters

**Figure 9:**
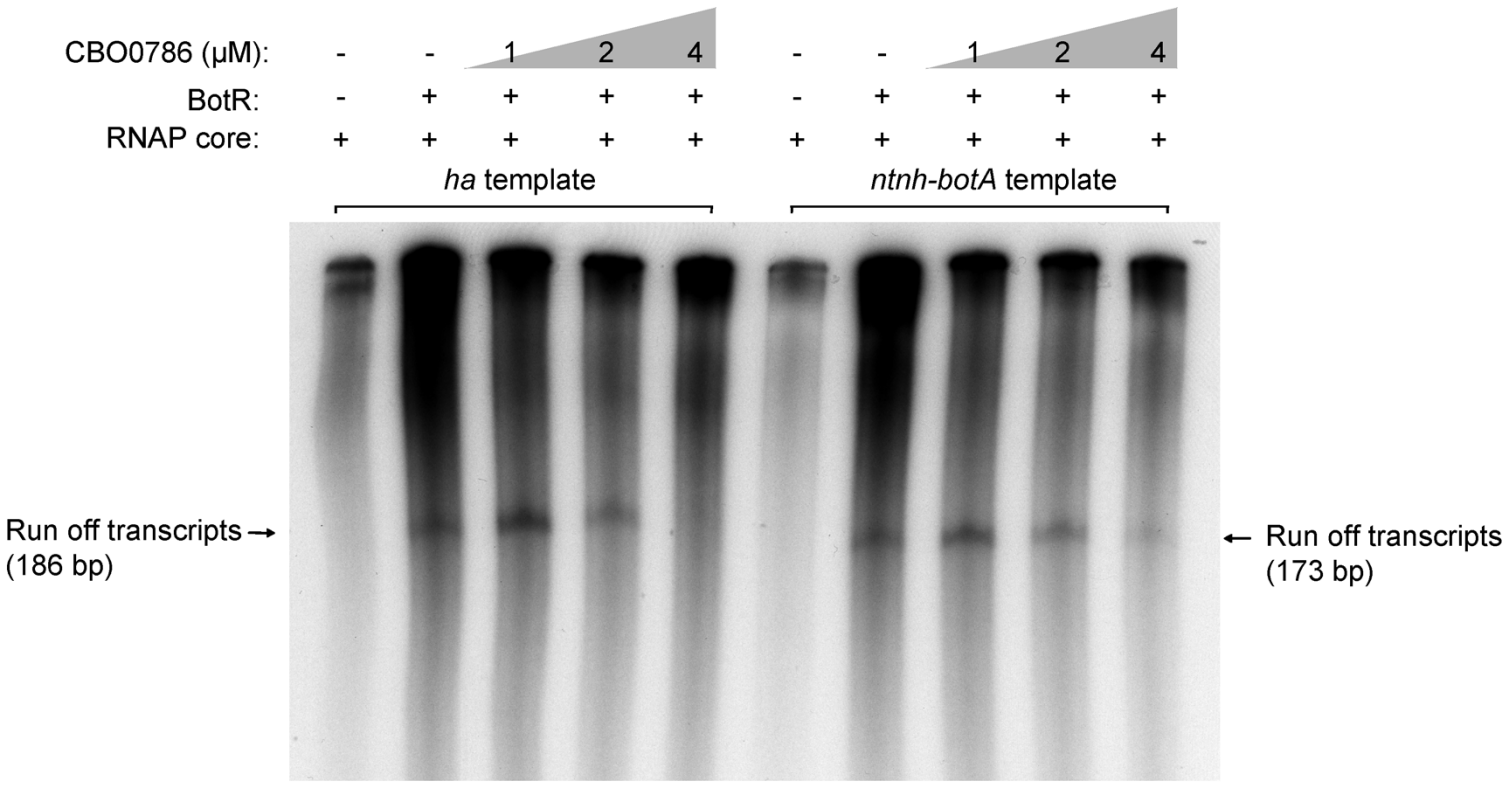
CBO0786 represses transcription from *ha* and *ntnh*-*botA* promoters *in vitro*. Run-off transcription from *ha33* and *ntnh-botA* promoter-containing templates was repressed in the presence of increasing concentration of CBO0786. Arrow indicates the run-off transcripts.

To demonstrate the direct effect of CBO0786 on transcription of the neurotoxin genes, we carried out *in vitro* run-off transcription assays using RNAP reconstituted with *E. coli* RNAP core enzyme and the purified sigma factor BotR (Figure 9). DNA fragments containing the promoter of the *ha* or *ntnh-botA* operons were cloned and used as transcription templates. As expected, transcripts produced from the promoters of *ha* or *ntnh-botA* operons were observed only in the presence of both RNAP core enzyme and BotR. Addition of increasing concentrations of recombinant CBO0786 caused gradual inhibition of both transcripts, suggesting CBO0786 directly represses the transcription from *ha* and *ntnh*-*botA* promoters. Transcription from the *ha* promoter was more efficiently repressed than that from the *ntnh-botA* promoter in the presence of 4 µM CBO0786 (Figure 9). Taken together, CBO0786 exhibits a higher binding affinity to the direct repeat of the −10 region (GTTATA) in the *ha* promoter than in the *ntnh-botA* promoter, and consequently shows a more effective inhibition of *in vitro* transcription from the *ha* promoter than from the *ntnh-botA* promoter. These results indicate that CBO0786 represses the transcription of neurotoxin gene cluster by binding to the consensus core promoter −10 region of *ha* and *ntnh-botA* operons.

To test whether CBO0786 represses the transcription of *botR*, we also performed *in vitro* run-off transcription assays with DNA template containing the *botR* promoter. No clear transcript from *botR* promoter was observed in the presence of RNAP core with BotR. Consistent with a previous study [20], the result demonstrates that *botR* is not auto-transcribed *in vitro*.

## Discussion

We show that the TCS CBO0787/CBO0786 negatively regulates botulinum neurotoxin expression in *C. botulinum* Group I type A1 strain ATCC 3502. This was supported by enhanced toxin gene expression and increased toxin synthesis by *cbo0787* and *cbo0786* mutants, by specific binding of recombinant CBO0786 regulator to the neurotoxin promoters, and recombinant CBO0786 inhibiting *in vitro* transcription from the neurotoxin promoters. Identification of botulinum neurotoxin repressors has been an unattainable target of neurotoxin research for decades [13], [36], [37], and would open up novel strategies for controlling the public health risks caused by the toxin, but also for enhancing industrial processes for therapeutic neurotoxin preparations.

While the current work focused on the most well-characterized *C. botulinum* type A1 neurotoxin subtype, the role of CBO0787/CBO0786 homologs in strains of other subtypes will be an interesting line of future research. While the *cbo0787*/*cbo0786* locus is found at an 11-kb distance from the neurotoxin gene cluster (*cbo0801*–*cbo0806*) in ATCC 3502, a TCS showing a strikingly high (>95%) amino acid identity to CBO0787/CBO0786 is similarly encoded by loci near the neurotoxin gene cluster in the genomes of type A2 strain Kyoto (3.6 kb distance), type A5 strain H04402 065 (12 kb), and type F strain Langeland (24 kb). This TCS, therefore, is an interesting candidate for a universal neurotoxin repressor in Group I *C. botulinum*.

Our biochemical data suggest that CBO0786 recognizes and binds to the consensus −10 region (GTTATA) of the *ha* and *ntnh*-*botA* operon promoters. The −10 region is also specifically recognized by the alternative sigma factor BotR [20], [38], suggesting that CBO0786 binding to the core promoter −10 region may prevent RNAP-BotR from binding and initiating the transcription. The *in vitro* run-off transcription assay demonstrated that CBO0786 directly inhibits RNAP-BotR-directed transcription from the *ha* and *ntnh*-*botA* promoters, supporting the hypothesis that CBO0786 inhibits the transcription of these operons.

BotR is specifically required for botulinum neurotoxin gene expression [25] but does not autoregulate its own expression [20]. While the −35 site (TTTACA) of the *ha* and *ntnh*-*botA* core promoters is also found upstream of *botR*, the −10 site of *botR* promoter is different (TTCGTA) [20]. Accordingly, we did not observe CBO0786 binding to *botR* promoter. Moreover, we did not find additional CBO0786 binding sites downstream of the *botR* promoter *in vitro* or *in silico*. Thus it is plausible that CBO0786 does not repress *botR* transcription.

While an early study detected both monocistronic *botA* and bicistronic *ntnh*-*botA* mRNA species and a putative transcription start site in the intergenic region between *ntnh* and *botA* [34], BotR was later shown to exclusively drive the bicistronic form of transcription [20]. Accordingly, we could not identify a CBO0786 binding site in the intergenic region between *ntnh* and *botA*, thus inhibition by CBO0786 of transcription from a putative *botA*-specific promoter is not likely.

BotR homologues in other pathogenic *Clostridia*, such as TetR controlling tetanus neurotoxin in *Clostridium tetani* [39], TcdR controlling toxins A and B in *Clostridium difficile* [40], and UviA controlling bacteriocins in *Clostridium perfringens* [41] all recognize the same −35 sequence as BotR [20]. Interestingly, TetR also recognizes the same −10 box (GTTATA) as BotR [36]. The closest homolog of CBO0787/CBO786 in *C. tetani* is CTC01420/CTC01421, with the response regulator CTC01421 showing 75% amino acid similarity to CBO0786. Thus CTC01420/CTC01421 may be an interesting candidate for a tetanus neurotoxin repressor.

Our qRT-PCR and ELISA analysis, albeit yielding significantly greater neurotoxin gene expression in both *cbo0787* and *cbo0786* mutants than in WT, suggest the mutant phenotypes to be somewhat less striking than reported for some well-characterized TCSs in other Gram-positive bacteria. For example, mutation of CsrS, reported to respond to environmental Mg^2+^ [42] and to repress virulence-related capsular polysaccharide production in group A *Streptococcus*, resulted in a 10-fold increase in cellular hyaluronic acid production in the presence of Mg^2+^ [42]. However, in medium lacking Mg^2+^, only a modest 1.5-fold induction in hyaluronic acid production was observed [42]. Thus the presence of the signal triggering a TCS kinase is a key to control the activity of the cognate response regulator and its subsequent effects on target gene expression. The signal triggering CBO0787 and the role of phosphotransfer between CBO0787 and CBO0786 remain to be elucidated. Although our EMSA analysis suggested a DNA-binding activity for CBO0786 both with and without phosphorylation *in vitro*, which is in line with reports on some other TCS response regulators showing similar DNA-binding properties regardless of phosphorylation state [43], *in vivo* phosphorylation of CBO0786 could lead to conformational changes that fine-tune its DNA binding affinity [44]–[46]. Identification of the signal triggering CBO0787 will be an important future task for optimization of the study conditions to detect maximal mutant phenotypes and thus to detect the maximal effect of CBO0786 on neurotoxin repression.

The sensor domain of CBO0787 is predicted to contain an extracellular loop consisting of 79 amino acid residues and is flanked by two transmembrane helices, indicating that the signal triggering it is most likely extracellular. Moreover, the increasing differences observed between the relative neurotoxin gene expression levels of the *cbo0787* kinase mutant and WT towards stationary growth phase are consistent with cell density-dependent signals [37]. Neurotoxin overproduction associated with inability to sporulate by strain Hall A-hyper [13] suggests that regulation of these processes may be linked. Repression of toxin synthesis after logarithmic growth before initiation of sporulation may represent a survival mechanism when nutrient sources become limited. Moreover, uncontrolled synthesis of botulinum neurotoxin would waste energy since only nanogram quantities are sufficient for *C. botulinum* to kill mammals and thereby gain nutrients and establish anaerobiosis.

Previous studies have identified excess of arginine [21] and tryptophan [22] to repress botulinum neurotoxin formation. By contrast, glucose was shown to induce neurotoxin synthesis [21]. Interestingly, glucose has also been linked with regulation of toxin synthesis in *C. difficile* through carbon catabolite control [47], [48]. Further research will be required to clarify the mechanisms by which nitrogen sources or glucose control neurotoxin synthesis in *C. botulinum*; however, our preliminary data do not indicate that these compounds trigger CBO0787 (data not shown). Thus it is plausible that the regulation of neurotoxin synthesis is accomplished through a complex network and other regulators are involved in this control.

While the relative neurotoxin gene expression levels in the *cbo0786* regulator mutant were significantly higher than those of the WT at logarithmic growth phase, this difference was smaller at early stationary growth phase. Moreover, the wild-type levels of *cbo0786* and *cbo0787* transcription, being stable throughout the logarithmic growth phase, collapsed at the transition into stationary phase. These observations further support the involvement of repressors other than CBO0786 in the ‘switching-off’ of the neurotoxin expression, ensuring efficient onset of stationary-phase cellular events.

Bearing in mind the emergence of repressor gene (*tcdC*) mutations in ‘hyper-virulent’ isolates of the notorious healthcare-associated pathogen *C. difficile* [49], [50], the emergence of *C. botulinum* strains with a mutated neurotoxin repressor would set challenges to public health and safety. Hence identification of neurotoxin regulators and their mutations is crucial.

In conclusion, we propose that the TCS CBO0787/CBO0786 negatively regulates botulinum neurotoxin gene transcription through the response regulator CBO0786 blocking the −10 core promoter sites of the *ha* and *ntnh*-*botA* operons, inhibiting transcription from the *ha* and *ntnh*-*botA* promoters. These data provide keys for controlling the production of botulinum neurotoxin, which is a major target of the food and pharmaceutical industries.

## Materials and Methods

### Strains, culture, plasmids, and oligonucoleotides

Bacterial strains and plasmids are described in Table S1. *C. botulinum* Group I type A1 strain ATCC 3502 [29] and derivative mutants were grown in anaerobic tryptone-peptone-glucose-yeast extract (TPGY) medium at 37°C under strictly anaerobic conditions. Cell counts were determined by plating serially diluted cultures on anaerobic TPGY agar plates. *Escherichia coli* conjugation donor CA434 [51] and *E. coli* TOP10 strain (Invitrogen) were grown in Luria-Bertani (LB) medium at 37°C. When appropriate, growth media were supplemented with 100 µg/ml ampicillin, 50 µg/ml kanamycin, 25 µg/ml chloramphenicol, 250 µg/ml cycloserine, 15 µg/ml thiamphenicol or 2.5 µg/ml erythromycin. All oligonucleotide primers are listed in Table S2.

### Mutation of *cbo0787* and *cbo0786*

Target genes were insertionally inactivated in *C. botulinum* ATCC3502 by using the ClosTron system as previously reported [33], in combination with the TargeTron gene knockout system kit (Sigma-Aldrich). Target sites in *cbo0786* (between nucleotides 267–268) and *cbo0787* (between nucleotides 603–604) were identified, and intron-retargeting PCR primers (Table S2) were designed by using the TargeTron algorithm (http://www.sigma-genosys.com/targetron/).

Plasmid retargeting was carried out as previously described [33] and the resulting plasmid pMTL007::*cbo0786* or pMTL007::*cbo0787* was transferred to *C. botulinum* ATCC3502 by conjugation from *E. coli* CA434 [51]. Successful transconjugants were screened on TPGY agar plates containing cycloserine (250 µg/ml) and thiamphenicol (15 µg/ml), and then resuspended in 1 ml of anaerobic TPGY containing 1 mM isopropyl-β-D-thiogalactopyranoside (IPTG) and thiamphenicol (7.5 µg/ml) and incubated at 37°C for 3 h. The bacteria were then harvested, resuspended in 1 ml of fresh TPGY and incubated for a further 3 h. The subsequent integrants were selected by plating bacteria on TPGY agar supplemented with erythromycin (2.5 µg/ml) and cycloserine (250 µg/ml) and incubated for 16 h at 37°C in anaerobic conditions to select clones harboring the spliced erythromycin retrotransposition activated marker (ErmRAM), which indicates intron integration.

To demonstrate the integration of the Ll.LtrB intron in the desired sites, PCR was performed using primers flanking the target sites in *cbo0786* and *cbo0787* (Table S2). PCR using ErmRAM primers demonstrated the spliced ErmRAM. In addition, to confirm that only a single intron insertion occurred in each mutant, genomic DNA from the ATCC3502 wild-type strain and mutants, and the pMTL007 plasmid DNA were digested overnight with HindIII and analysed by Southern blot probed with a DIG-labeled fragment derived from the Ll.LtrB intron sequence.

### Complementation of mutation

For complementation, a 2441-bp fragment encompassing *cbo0786*, *cbo0787*, and the 5′ noncoding region including their putative promoter, was cloned into plasmid pMTL82151 [52] to make pMTL::*cbo0787*/*0786*. pMTL::*cbo0787*/*0786* or pMTL82151 (empty-vector control) was transferred to *C. botulinum* ATCC3502 or *cbo0786* mutant by conjugation from *E. coli* CA434. The complementation strain of *cbo0786* mutant-pMTL::*cbo0787*/*0786* and control strains of *C. botulinum* ATCC3502-pMTL and *cbo0786* mutant-pMTL were obtained.

### RNA isolation and qRT-PCR

Total RNA from *C. botulinum* ATCC 3502 and the two mutants was isolated using the RNeasy Mini Kit (Qiagen) as described [31]. Residual DNA was removed sequentially with RNase-free DNase set (Qiagen) and the DNA-free Kit (Ambion) according to the manufacturers' instructions. The RNA was dissolved in 50 µl of nuclease-free water (Sigma-Aldrich) and its concentration was determined using the NanoDrop ND1000 spectrophotometer (NanoDrop Technologies). The integrity of RNA was confirmed with the Agilent Technologies 2100 Bioanalyzer.

For qRT-PCR, samples were collected during mid-exponential, late exponential and early stationary growth phases (Figure 2). Duplicate cDNA samples were generated from 800 ng of RNA using the DyNAmo cDNA Synthesis Kit (Finnzymes). Quantitative real-time PCR reactions comprised of 1× DyNAmo Flash SYBR Green I Master Mix (Finnzymes), 0.5 mM of each primer (Table S2), and 4 µl of 10^2^-fold (*cbo0786*, *cbo0787*, *botA*, *ha33*) or 10^5^-fold (16S *rrn*) diluted cDNA template in a total volume of 20 ul. All PCRs were performed in duplicate for both cDNA replicates and three replicated experiments. Real-time PCR was performed using the Rotor-Gene 3000 real-time thermal cycler (Corbett Life Science). Cycling conditions included 7 minutes at 95°C, followed by 45 cycles of 95°C for 10 seconds and 60°C for 20 seconds, followed by 30 seconds at 60°C. PCR efficiencies were determined based on a standard curve made from serially diluted pooled cDNA for each primer pair. The calculated efficiencies were 0.97 for 16S *rrn*, 0.99 for *botA*, 0.92 for *ha33*, 1.04 for *cbo0787* and 0.93 for *cbo0786*. Melting curve analysis was performed following the completion of the PCR to confirm specificity of the PCR amplification products. Target gene expression was normalized to the expression of 16S *rrn* based on the Pfaffl method [53]. All samples were calibrated against the wild type culture at mid-exponential growth phase.

### Neurotoxin ELISA

Botulinum neurotoxin was quantified by using a commercial type A neurotoxin ELISA kit (Tetracore) in three independent WT and mutant culture supernatants collected at mid-exponential, late exponential, and early stationary growth phases [24], [26], [28]. The plates were read at 405 nm (Multiskan Ascent, Thermo Fisher). To keep the optical density readings in the dynamic range, the culture supernatants were diluted 1∶10 at mid-exponential, 1∶20 at late exponential, and 1∶30 at early stationary growth phase.

### Expression and purification of recombinant CBO0786 and BotR

To construct the plasmids for the expression of N-terminal 6-histidine translation fusion to the response regulator CBO0786 or the alternative sigma factor BotR, PCR products were generated using the primers listed in Table S2. The PCR products of *cbo0786* and *botR* were digested with appropriate restriction enzymes and cloned individually into plasmid pET28b (Novagen). The plasmids were then individually transformed into *E. coli* Rosetta 2(DE3) pLysS cells (Novagen).

CBO0786 expression was induced with 1 mM IPTG at 37°C for 5 h. Cells from a 500-ml culture were harvested, re-suspended in 10 ml of lysis/binding buffer (500 mM NaCl, 20 mM imidazole, 20 mM Tris-HCl, pH 7.9) and lysed by sonication. The lysate was centrifuged at 10 000 g for 15 min and filtered through a 0.45-µm filter. The lysate was loaded with 1 ml of Novagen His Bind affinity resin and allowed to bind for 30 min at 4°C. The resin was washed by 10 ml of lysis/binding buffer and 20 ml of wash buffer (500 mM NaCl, 60 mM imidazole, 20 mM Tris-HCl, pH 7.9). Bound protein was eluted by washing with 4 ml of elution buffer (500 mM NaCl, 500 mM imidazole, 20 mM Tris-HCl, pH 7.9).

BotR expression was induced with 1 mM IPTG overnight at 20°C and purified as described previously [20], with some modifications. Briefly, cells were lysed by sonication in lysis/binding buffer. The insoluble cell debris was separated by centrifugation and dissolved in denaturing lysis/binding buffer with 6 M guanidine hydrochloride. After centrifugation and filtration, the denatured solubilized proteins were collected and loaded onto column containing Ni-NTA affinity resin. The bound proteins were let to refold on column with a decreasing urea gradient (6 to 0 M) in lysis/binding buffer. Finally, the refolded proteins were obtained by washing with elution buffer.

Eluted fractions were examined by SDS-PAGE and fractions containing CBO0786 or BotR were pooled in the Novagen D-tube Dialyzer and dialysed against 1 l of dialysis buffer (300 mM NaCl, 20% glycerol, 50 mM Tris-HCl, pH 8.0) overnight at 4°C. Protein concentrations were determined by using the Bradford reagent (Bio-Rad) and BSA (Sigma-Aldrich) was used as a standard.

### EMSA

A 354-bp fragment (P*ha33* probe) covering the intergenic region between *ha33* and *botR* (−287 bp to 67 bp of *ha33*) and a 262-bp fragment (P*ntnh-botA* probe) covering the intergenic region between *botR* and *ntnh* (−210 bp to 52 bp of *ntnh*) were amplified by PCR using 5′-end biotin labeled primers (Table S2). CBO0786 phosphorylation by acetyl phosphate was performed as described [54]. EMSA was performed with 1 nM of 5′-end biotin labeled, double-stranded oligonucleotide probes, 0 to 5.4 µM of recombinant CBO0786, 1 µg of poly(dI-dC), 2.5% glycerol and 5 mM MgCl_2_ in binding buffer (LightShift Chemiluminescent EMSA Kit, Pierce). For competition assays, a 200-fold molar excess of unlabeled double-stranded oligonucleotide was added. Binding was allowed to proceed for 30 min at room temperature. Band shifts were resolved on a 5% native polyacrylamide gel run in 0.5× TBE at 4°C for 1 h at 110 V.

### DNase I footprinting

DNase I footprinting was performed in triplicate using a modification of [55]. The P*ha33* and P*ntnh-botA* probes were amplified by PCR using 6-FAM-labeled forward primers and HEX-labeled reverse primers (Table S2). Binding reactions were performed as described for EMSA, with 20 nM of 5′-6-FAM-labeled probe and 10 µM protein in a final volume of 20 µl. After 20 min of incubation, DNA probes were partially digested by 0.002 to 0.2 Kunitz unit of DNase I (Sigma-Aldrich) for 5 min at room temperature. Reactions were stopped by addition of 22 µl of 0.5 M EDTA and heated at 70°C for 10 min. The digested DNA fragments were purified with the QIAquick PCR Purification Kit (Qiagen) and eluted in 25 µl of water. The purified fragments were separated in a capillary DNA analyzer (Applied Biosystems 3130×l DNA Analyzer) and the electropherograms were analyzed using the Peak Scanner software (Applied Biosystems). Protected regions were identified by sequencing of the fragments using the Thermo Sequenase Cycle Sequencing Kit (Affymetrix) and the same labeled primers as described above (Table S2).

### *In vitro* run-off transcription

The upstream region of the *ha* operon spanning the −191 to +185 sites relative to the transcription start, and the upstream region of the *ntnh-botA* operon spanning the −108 to +172 sites relative to the transcription start, were cloned into pBluescript II KS- (Stratagene) and then linearized by SpeI or PstI to produce the run-off transcription templates. *In vitro* transcription assays were carried out in 10-µl reaction mixtures, in the absence or presence of CBO0786. *E. coli* RNAP core enzyme (Epicentre) was preincubated for 30 min at 37°C with six-fold molar excess of purified BotR. After incubation, 0.5 U RNAP, 0 to 4 µM of recombinant CBO0786, and 15 nM of linearized plasmid DNA were added in transcription buffer containing 40 mM Tris-HCl, pH 8.0, 10 mM MgCl_2_, 50 mM KCl, 0.1 mg/ml BSA, 5% glycerol, 4 U of RNasin (Promega), and incubated for 10 min at 37°C. Transcription was initiated by the addition of 200 µM each of ATP, GTP and CTP, 50 µM of non-radioactive UTP and 2.5 µCi [α-^32^P]-UTP (3000 Cimmol^−1^, Perkin-Elmer). After further incubation for 30 min at 37°C, the reaction was quenched by adding 10 µl of RNA loading buffer (95% formamide, 0.025% bromophenol blue, 0.025% xylene cyanol FF, and 5 mM EDTA), followed by heating for 10 min at 80°C. Samples were resolved by denaturing 6% PAGE and visualized by autoradiography.

### Statistical analysis

For any pairwise comparisons between the WT and one of the two mutant strains, Student's t-test was used. For a multiple comparison among the *cbo0786*-pMTL::*cbo0787*/*0786*, *cbo0786*-pMTL, and WT-pMTL, one-way ANOVA with Tukey's post hoc test was used.

### The NCBI ID numbers of the genes and genome studied

*cbo0787* (ID number 5185042), *cbo0786* (ID number 5185041), *cbo0803*, *ha33* (ID number 5185058), *cbo0805*, *ntnh* (ID number 5185060), *cbo0806*, *botA* (ID number 5185061), *Clostridium botulinum* type A strain ATCC 3502 genome (Accession number NC_009495.1).

## Supporting Information

Table S1 **Bacterial strains and plasmids.**
(DOC)

Table S2 **Oligonucleotides.**
(DOC)
