# Supplementary material for: Two-Component Signal Transduction System CBO0787/CBO0786 Represses Transcription from Botulinum Neurotoxin Promoters in Clostridium botulinum ATCC 3502
Source: PLoS Pathog. 2013 Mar 28;9(3):e1003252. doi: 10.1371/journal.ppat.1003252 (PMC3610760; doi:10.1371/journal.ppat.1003252)
Supplement: Table S1 — Bacterial strains and plasmids. (DOC) [file ppat.1003252.s001.doc]

Table S1. Bacterial strains and plasmids.

| Bacterial strain or plasmid | Relevant characteristics* | Reference or source |
| --- | --- | --- |
| *C. botulinum* strains |  |  |
| ATCC3502 | Isolated in 1940, producing type A1 neurotoxin | (1) |
| *cbo0786* mutant | ATCC3502 derivative, cbo0786 inserted with Ll.LtrB group II intron at the sense site of 267 bp, Em^R^ | This study |
| *cbo0787* mutant | ATCC3502 derivative, *cbo0787* inserted with Ll.LtrB group II intron at the antisense site of 603 bp, Em^R^ | This study |
| WT-pMTL | *C. botulinum* ATCC3502 carrying plasmid pMTL82151 | This study |
| *cbo0786*-pMTL | *C. botulinum* *cbo0786* mutant carrying plasmid pMTL82151 | This study |
| *cbo0786*-pMTL::*cbo0787*/*0786* | *C. botulinum* *cbo0786* mutant carrying complement plasmid pMTL::*cbo0787*/*0786* | This study |
| *E. coli strains* |  |  |
| Top10 | General cloning and blue/white screening | Invitrogen |
| CA434 | *E. coli* HB101 carrying the Incβ conjugative plasmid R702 | (2) |
| CA434-pMTL | *E. coli* CA434 carrying plasmid pMTL82151 | This study |
| CA434-pMTL::*cbo0787*/*0786* | *E. coli* CA434 carrying plasmid pMTL::*cbo0787*/*0786* | This study |
| Rosetta 2(DE3) pLysS | F^–^ *ompT hsdS*_B_(r_B_^–^ m_B_^–^) *gal dcm* (DE3) pLysSpRARE2^3^ (Cam^R^) | Novagen |
| Rosetta 2(DE3) pLysS-pET 28b::*cbo0786* | Rosetta 2(DE3) pLysS carrying plasmid pET 28b::*cbo0786* | This study |
| Rosetta 2(DE3) pLysS-pET 28b::*botR* | Rosetta 2(DE3) pLysS carrying plasmid pET 28b::*botR* | This study |
| Plasmid |  |  |
| pMTL007 | Inducible clostridial expression vector for expression of ClosTron, containing Erm RAM, ColE1, pCB102, Cm^R^ | (3) |
| pMTL007::*cbo0786*-267s | pMTL007 containing intron retargeted to *cbo0786* (sense insertion at 267 bp) | This study |
| pMTL007::*cbo0787*-603a | pMTL007 containing intron retargeted to *cbo0787* (antisense insertion at 603 bp) | This study |
| pMTL82151 | *Clostridium-E.coli* shuttle plasmid; pBP1 replicon; Cm^R^ | (4) |
| pMTL::*cbo0787*/*0786* | pMTL82151 containing the DNA fragment amplified by PCR using primers *cbo786*-F-NotI and *cbo0787*-R-NheI | This study |
| pET 28b | P_T7_, Kan^R^, *ori* pBR322, *ori* f1, *lac*I, T7 Tag N-terminal 6xHis, C-terminal 6xHis | Novagen |
| pBluescript II KS - | Cloning vector, T3 and T7 promoters, lacZ, pMB1 replicon; Amp^R^ | Stratagene |
| PET 28b::*cbo0786* | pET28b containing the DNA fragment amplified by PCR using primers *cbo0786*-F-NdeI and *cbo0786*-R-XhoI | This study |
| PET 28b::*botR* | pET28b containing the DNA fragment amplified by PCR using primers *botR*-F-NdeI and *botR*-R-XhoI | This study |

* Amp, ampicillin; Cm, chloramphenicol; Em, erythromycin; R, resistant.

**References**

1. Sebaihia M, Peck MW, Minton NP, Thomson NR, Holden MT, Mitchell WJ, Carter AT, Bentley SD, Mason DR, Crossman L, Paul CJ, Ivens A, Wells-Bennik MH, Davis IJ, Cerdeño-Tárraga AM, Churcher C, Quail MA, Chillingworth T, Feltwell T, Fraser A, Goodhead I, Hance Z, Jagels K, Larke N, Maddison M, Moule S, Mungall K, Norbertczak H, Rabbinowitsch E, Sanders M, Simmonds M, White B, Whithead S, Parkhill J (2007) Genome sequence of a proteolytic (Group I) *Clostridium botulinum* strain Hall A and comparative analysis of the clostridial genomes. *Genome Res* 17:1082-1092.
2. Purdy D, O’Keeffe TAT, Elmore M, Herbert M, McLeod A, Bokori-Brown M, Ostrowski A, Minton NP (2002) Conjugative transfer of clostridial shuttle vectors from Escherichia coli to Clostridium difficile through circumvention of the restriction barrier. Mol Microbiol 46:439-452.
3. Heap JT, Pennington OJ, Cartman ST, Carter GP, Minton NP (2007) The ClosTron: a universal gene knock-out system for the genus *Clostridium*. *J Microbiol Methods* 70:452-464.
4. Heap JT, Pennington OJ, Cartman ST, Minton NP (2009) A modular system for *Clostridium* shuttle plasmids. *J Microbiol Methods* 78:79-85.
