# Supplementary material for: Two-Component Signal Transduction System CBO0787/CBO0786 Represses Transcription from Botulinum Neurotoxin Promoters in Clostridium botulinum ATCC 3502
Source: PLoS Pathog. 2013 Mar 28;9(3):e1003252. doi: 10.1371/journal.ppat.1003252 (PMC3610760; doi:10.1371/journal.ppat.1003252)
Supplement: Table S2 — Oligonucleotides. (DOC) [file ppat.1003252.s002.doc]

Table S2. Oligonucleotides.

| Primer | Sequence(5’-3’) | |
| --- | --- | --- |
| **ClosTron mutagenesis** | | |
| *cbo0786*-IBS | AAAAAAGCTTATAATTATCCTTAAATGTCGTTTTAGTGCGCCCAGATAGGGTG | |
| *cbo0786*-EBS1d | CAGATTGTACAAATGTGGTGATAACAGATAAGTCGTTTTAGGTAACTTACCTTTCTTTGT | |
| *cbo0786*-EBS2 | TGAACGCAAGTTTCTAATTTCGGTTACATTCCGATAGAGGAAAGTGTCT | |
| *cbo0787*-IBS | AAAAAAGCTTATAATTATCCTTATATCTCATATTAGTGCGCCCAGATAGGGTG | |
| *cbo0787*-EBS1d | CAGATTGTACAAATGTGGTGATAACAGATAAGTCATATTATGTAACTTACCTTTCTTTGT | |
| *cbo0787*-EBS2 | TGAACGCAAGTTTCTAATTTCGGTTAGATATCGATAGAGGAAAGTGTCT | |
| EBS Universal | CGAAATTAGAAACTTGCGTTCAGTAAAC | |
| **PCR screening** | | |
| *cbo0786*-40-F | GCCATTGGAGTGGAATATAC | |
| *cbo0786*-446-R | GGCTCTATTATTATGTCCTTGG | |
| *cbo0786*-Spec-F | ATATTTATGACTGCCTGTG | |
| *cbo0787*-429-F | AGCGGAAGTGTTTACTAAAG | |
| *cbo0787*-948-R | TTCTGCTGTCCAATCCATAT | |
| *cbo0787*-Spec-R | ATTCCACTACTCCAGCCTC | |
| **Southern blots** | | |
| intron-693-F | GCGTGCGACTCATAGAAT | |
| intron-1045-R | TTGGATATTCACCGAACACT | |
| **qRT-PCR** | | |
| *botA*-F | CGCGAAATGGTTATGGCTCT | |
| *botA*-R | GCCTGCACCTAAAAGAGGATTT | |
| *ha33*-F | CATCTCCTGTAAGGCCGATACTAA | |
| *ha33*-R | GCATTTGAATCTTGTTGCGTTG | |
| 16S*rrn*-F | AGCGGTGAAATGCGTAGAGA | |
| 16S*rrn*-R | GGCACAGGGGGAGTTGATAC | |
| **Complement of *cbo0786* mutant** | | |
| *cbo0786*-F-NotI | NNNNNNGCGGCCGCTAATTAAGTTTACAAGGATTCATAGCTCAC | |
| *cbo0787*-R-NheI | NNNNNNGCTAGCGAATGAACCATCTTCACTATTAA | |
| **Protein expression** | | |
| *cbo0786*-F-NdeI | NNNNNNNNCATATGACAAAAATATTATTAGTAGAAGATGATATGGCT | |
| *cbo0786*-R-XhoI | NNNNNNNNCTCGAGTTACTCACTCCACCTATAGCCTATTCC | |
| *botR*-F-NdeI NNNNNNCATATGAATAAATTGTTTTTACAAATTAAAATGTT | | |
| *botR*-R-XhoI NNNNNNCTCGAGCTACATATTAATTAATTTTTTCAATATGGG | | |
| **EMSA** | | |
| *ntnh*-F | (5’-end labelled biotin)GGCTTTAGAGAGATTAGAACCCATA | |
| *ntnh*-R | (5’-end labelled biotin)CATTTTTATTATCTACCGGGGAAT | |
| *ha33*-F | (5’-end labelled biotin)CGGCCTTACAGGAGATGGTA | |
| *ha33*-R | (5’-end labelled biotin)TTTCTTGAAACTCCCTATTGTCA | |
| **DNase I footprinting** | | |
| *ntnh*-F | (5’-end labelled 6-FAM)GGCTTTAGAGAGATTAGAACCCATA | |
| *ntnh*-R | (5’-end labelled HEX)CATTTTTATTATCTACCGGGGAAT | |
| *ha33*-F | (5’-end labelled 6-FAM)CGGCCTTACAGGAGATGGTA | |
| *ha33*-R | (5’-end labelled HEX)TTTCTTGAAACTCCCTATTGTCA | |
| ***In vitro* run-off transcription** | |  |
| *ha33*-SpeI GCGGACTAGTAGTCTCCATCTTTCAAGGTA | |  |
| *ha33*-PstI GCGGCTGCAGTTTACCACTACCCTTCCATA | |  |
| *ntnh*-PstI CGGCTGCAGGCTTTAGAGAGATTAGAACCC | |  |
| *ntnh*-SpeI GCGGACTAGTCTAGCTCTAACTACTACAAC | |  |
